# Doxorubicin Conjugated to Glutathione Stabilized Gold Nanoparticles (Au-GSH-Dox) as an Effective Therapeutic Agent for Feline Injection-Site Sarcomas—Chick Embryo Chorioallantoic Membrane Study

**DOI:** 10.3390/molecules22020253

**Published:** 2017-02-08

**Authors:** Katarzyna Zabielska-Koczywąs, Izabella Dolka, Magdalena Król, Artur Żbikowski, Wiktor Lewandowski, Józef Mieczkowski, Michał Wójcik, Roman Lechowski

**Affiliations:** 1 Faculty of Veterinary Medicine, Warsaw University of Life Sciences, Nowoursynowska 166, 02-776 Warsaw, Poland; izabella_dolka@sggw.pl (I.D.); magdalena_krol@sggw.pl (M.K.); artur_zbikowski@sggw.pl (A.Ż.); roman_lechowski@sggw.pl (R.L.); 2 Faculty of Chemistry, University of Warsaw, Pasteura 1, 02-093 Warsaw, Poland; wlewandowski@chem.uw.edu.pl (W.L.); mieczkow@chem.uw.edu.pl (J.M.); mwojcik@chem.uw.edu.pl (M.W.)

**Keywords:** in vivo study, doxorubicin, gold nanoparticles, CAM assay, feline injection-site sarcoma, Ki-67

## Abstract

Feline injection-site sarcomas are malignant skin tumours with a high local recurrence rate, ranging from 14% to 28%. The treatment of feline injection-site sarcomas includes radical surgery, radiotherapy and/or chemotherapy. In our previous study it has been demonstrated that doxorubicin conjugated to glutathione-stabilized gold nanoparticles (Au-GSH-Dox) has higher cytotoxic effects than free doxorubicin for feline fibrosarcoma cell lines with high glycoprotein P activity (FFS1, FFS3). The aim of the present study was to assess the effectiveness of intratumoural injection of Au-GSH-Dox on the growth of tumours from the FFS1 and FFS3 cell lines on chick embryo chorioallantoic membrane. This model has been utilized both in human and veterinary medicine for preclinical oncological studies. The influence of intratumoural injections of Au-GSH-Dox, glutathione-stabilized gold nanoparticles and doxorubicin alone on the Ki-67 proliferation marker was also checked. We demonstrated that the volume ratio of tumours from the FFS1 and FFS3 cell lines was significantly (*p* < 0.01) decreased after a single intratumoural injection of Au-GSH-Dox, which confirms the positive results of in vitro studies and indicates that Au-GSH-Dox may be a potent new therapeutic agent for feline injection-site sarcomas.

## 1. Introduction

Feline injection-site sarcomas (FISS) are malignant skin tumours with a high local recurrence rate (14%–28%) [1] and metastasis risk ranging from 10% to 28% [2]. Radical surgery with at least 5 cm margin of safety tissue is a first-line treatment for FISS [3], however, it is insufficient as monotherapy, therefore adjunct treatment—radiotherapy and/or chemotherapy—is recommended [4]. Both radiotherapy and chemotherapy may be used as a palliative treatment or as a neoadjuvant therapy in order to decrease the tumour size to facilitate the use of radical surgery. However, radiotherapy for animals is still unavailable in many countries [5]. On the other hand, the use of standard cytostatic treatment is limited due to the adverse side effects, high toxicity, nonspecific biodistribution, low therapeutic index, low water solubility and multidrug resistance (MDR) [6]. Gold nanoparticles have been proven to avoid glycoprotein P (P-gp)—the main efflux pump responsible for MDR—by entering the neoplastic cells through endocytosis [7,8]. In previously performed in vitro studies we have proven that doxorubicin conjugated to glutathione stabilized gold nanoparticles (Au-GSH-Dox) has a higher cytotoxic effect for feline fibrosarcoma cell lines (FFS1, FFS3) with high P-glycoprotein (P-gp) activity [9]. Nevertheless, further in vivo studies were needed to confirm this hypothesis. As a result, the objective of this study was to assess the anti-tumour efficacy of Au-GSH-Dox for FISS using the chick embryo chorioallantoic membrane (CAM) model. The CAM model has been successfully utilized for various biological, bioengineering and medical studies. It has been utilized for preclinical oncological studies in human and veterinary medicine as a cost-effective and easy to implement model. Despite its obvious limitations compared to in vivo studies on rodent models (e.g., the impossibility to perform pharmacokinetic studies) it is a good model for general anticancer compound screening because it allows for a reduction in the number of animals used for further in vivo studies according to 3R rule. It may therefore be used as an intermediate model between in vitro and in vivo studies [5,10,11,12,13,14]. It has been successfully used for assessing tumour progression [13], cancer cell invasion and metastasis [11,12,15], pro- and antiangiogenic drug response [16], vascular effects of photodynamic therapy [17], toxicological and efficiency studies on anticancer drugs [13,18,19,20,21].

## 2. Results and Discussion

**Figure 1:**
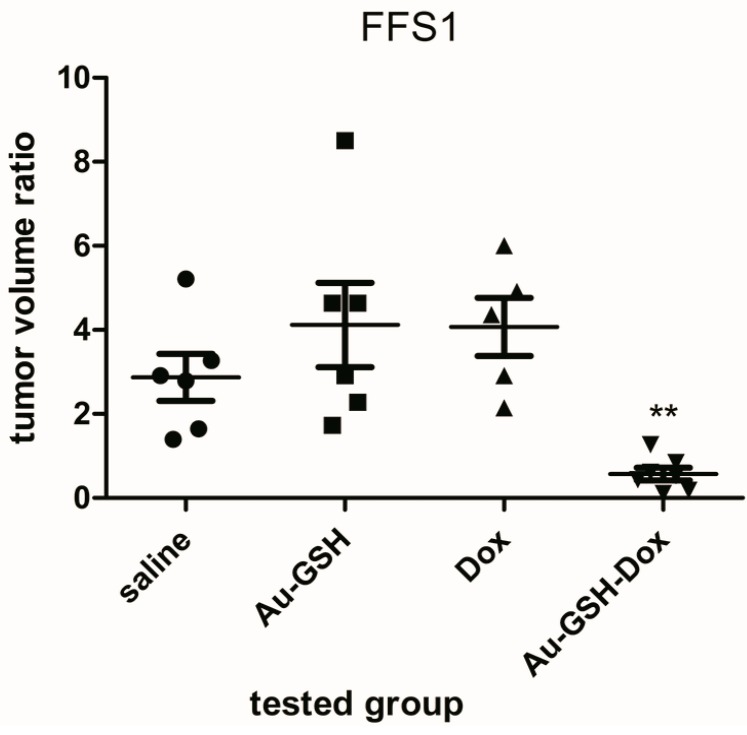
Volume ratio of tumours grown from the FFS1 cell line 72 h after a single intratumoural injection of the following compounds: saline (as a control) (●), Au-GSH (■), Au-GSH-Dox (▼), Dox (▲) compared to the tumour volume before treatment. ** *p* < 0.01 was assigned as highly significant.

**Figure 2:**
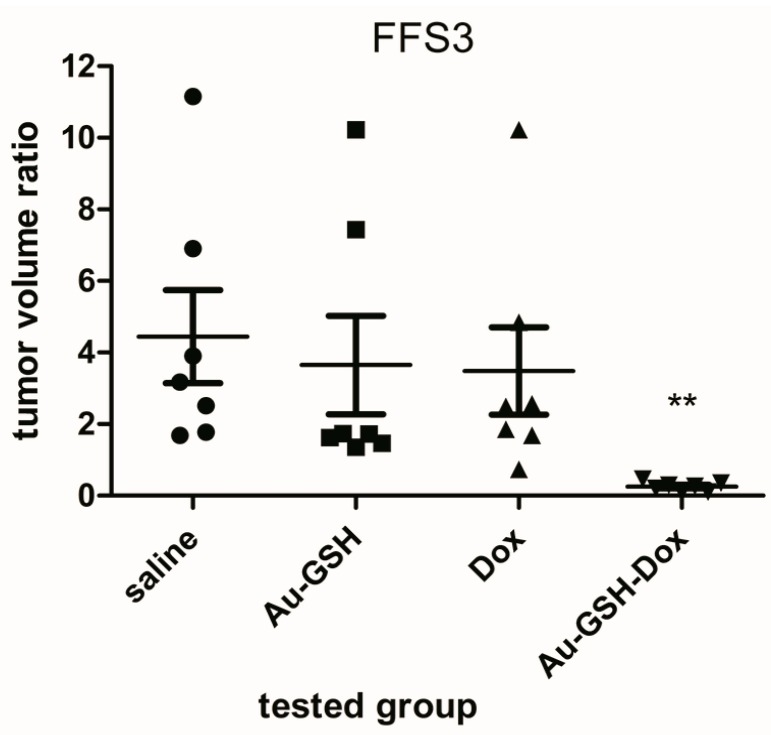
Volume ratio of tumours grown from the FFS3 cell line 72 h after single intratumoural injection of the following compounds: saline (●), Au-GSH (■), Au-GSH-Dox (▼), Dox (▲) compared to the tumour volume before treatment. ** *p* < 0.01 was assigned as highly significant.

The volume of all tumours grown from FFS1 and FFS3 cell lines was enlarged 72 h after a single intratumoural injection of both saline and Au-GSH. The average volume ratios for tumours from the FFS1 cell line were 2.62 and 3.75 after saline and Au-GSH injection, respectively (Figure 1). The average tumour volume ratios for tumours from the FFS3 cell line were 4.44 and 3.65 after saline and Au-GSH injection, respectively (Figure 2). There were no significant differences between the tumour volume ratio after injections of Dox, Au-GSH and saline, which indicates that doxorubicin is inefficient at the tested dose. The tumour volume ratios after the injection of Dox for tumours from the FFS1 and FFS3 cell lines were 5.58 and 3.39, respectively. On the other hand, there was a highly significant reduction in tumour volume ratio after a single Au-GSH-Dox injection (*p* < 0.01) (Figure 1 and Figure 2). The average tumour volume ratio after the injection of Au-GSH-Dox for tumours from the FFS1 cell line was 0.57 (six out of seven tumours decreased) (Figure 1) and for tumours from the FFS3 cell line it was 0.27 (all tumours decreased) (Figure 2). The results obtained in the presented in vivo studies indicate that intratumoural injections of Au-GSH-Dox may be effective in FISS treatment.

**Figure 3:**
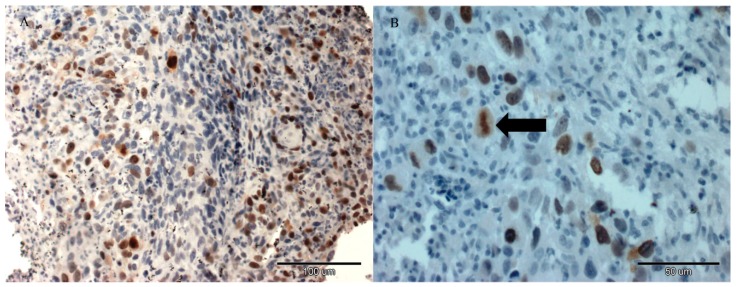
A positive Ki-67 expression was detected in the nuclei of tumour cells of feline injection-site sarcoma grown from the FFS3 cell line after a single intratumoural injection of Au-GSH-Dox. Scale bar = 100 μm (**A**), the arrow indicates a mitotic figure staining for Ki-67. Scale bar = 50 μm (**B**).

**Figure 4:**
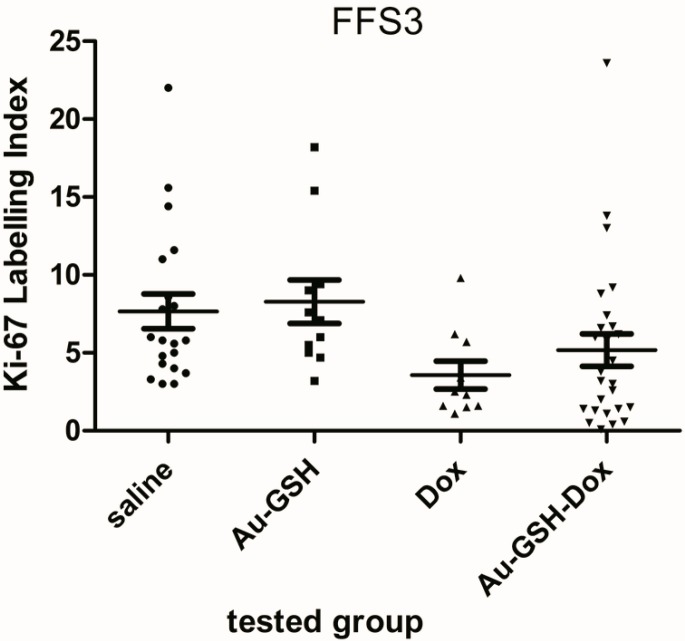
Ki-67 expression for tumours grown from the FFS3 cell line after single intratumoural injection of the following compounds: saline (as a control) (●), Au-GSH (■), Au-GSH-Dox (▼), Dox (▲) alone.

Fibrosarcomas grown from FFS1 cell lines showed no expression of Ki-67 in tumours from all tested groups. Fibrosarcomas grown from the FFS3 cell line had low Ki-67 expression (Figure 3) (ranging between 0% and 23.6%) in tumours from all tested groups, however, there were no statistically significant differences in Ki-67 expression in tumours after the injection of saline, Au-GSH, Au-GSH-Dox and Dox alone (Figure 4).

This indicates that a single intratumoural injection of Au-GSH, Au-GSH-Dox and Dox has no influence on the Ki-67 proliferation marker. In the presented study we tested the efficacy of intratumoural injections of Au-GSH-Dox. It has been recently demonstrated that intratumoural injections of cytotoxic drugs encapsulated into liposomes or conjugated to metal nanoparticles decrease the negative side effects of these drugs [22,23,24,25,26,27,28]. In human fibrosarcoma it was proven in vivo that intratumoural injections of liposomal doxorubicin inhibit tumour growth and prolong the overall survival of mice, in comparison to mice treated with doxorubicin alone. The concentration of liposomal doxorubicin was 5-times higher than free doxorubicin after intratumoural injection, which was probably due to liposomal doxorubicin entering the neoplastic cells through endocytosis. Moreover, the release of doxorubicin from the liposomes was proven to be slow and well-controlled [23]. 

The successful non-covalent attachment of doxorubicin to the glutathione-stabilized gold nanoparticles used has been analyzed using Fourier Transform Infrared Spectroscopy (FT-IR) and published in our previous paper [9]. The spectra of Au-GSH-Dox show characteristic additional bands (at 1280, 1404 and 1612 cm^−1^) due to Dox conjugation. To calculate the number of ligands on the nanoparticles’ surface, high-resolution X-ray photoelectron spectroscopy spectra was collected. Based on the relative areas and positions of the peaks the elemental composition of the obtained materials confirms ca. 85% of the Dox used for Au-GSH modification was present and bound with the nanoparticles’ surface [9]. Measurements on the cluster composition were conducted with a combination of analytical tools: high-resolution transmission electron microscopy (HRTEM), small angle X-ray scattering (SAXS) and X-ray photoelectron (XPS). The results from these various techniques are remarkably consistent and show each gold cluster is covered with 371 primary surfactant molecules (glutathione) and non-covalently attached 54 doxorubicin hydrochloride molecules. Non-covalently attached doxorubicin to gold nanoparticles has good penetration into the tumour tissue and it enables transport of the drug in an active form [29]. If the drug is attached covalently, it is transported in an inactive form and its release in the targeted tissue must be activated by internal or external factors. Huang et al. [30] proved that intratumoural injection of gold nanoparticles does not cause necrosis. Moreover, in a recent study on nude mice it was demonstrated that intratumourally injected doxorubicin conjugated to gold nanoparticles is efficient for human melanoma [28]. Similarly in the presented in vivo study on the chick embryo chorioallantoic membrane model we demonstrated that Au-GSH-Dox is efficient for treatment of FISS (Figure 1 and Figure 2). Also no necrosis was observed at the site of Au-GSH-Dox injections.

Although intratumoural injection of chemotherapeutic agents is not routinely used in clinical practise, intratumoural injection of carrier-based chemotherapy (e.g., HPMA copolymer-bound doxorubicin or ultra-small gold nanoparticles conjugated to doxorubicin) has recently been proposed as an alternative to routinely intravenous or oral administration [24,28]. Zhang and collaborators demonstrated that intratumoural injection of ultra-small gold nanoparticles conjugated to doxorubicin (Au-Dox) for accessible skin cancers (e.g., melanoma) may represent a viable approach for doxorubicin-resistant solid tumours as it reduces the risk of systemic toxicity. The main advantages of intratumoural administration are the higher concentration of anticancer agent at the target site, and lowering of the adverse side effects on the healthy tissue. Carmustine-containing polymeric wafers designed specifically for intraoperative intracerebral administration have been accepted by the Food and Drug Administration for treatment of glioblastoma, as they improve both efficacy and tolerability of the drug. Au-GSH-Dox presented in this study for further translational research may decrease the tumours’ size to enable surgical removal with a 5 cm margin of safety tissue or it may be used as palliative therapy in patients in whom standard therapy (surgery and radiotherapy) cannot be performed.

The CAM model has several advantages, such as a relatively simple experimental approach, low cost and fast tumour growth (tumours are visible within 3–5 days or 10 days after cancer cell implantation for human and animal cell lines, respectively). Moreover, it follows the “3R rule” (replacement, reduction and refinement) allowing reduction of the number of animals used in experiments, making the CAM model a promising intermediate model between in vitro and in vivo mammalian models. It could be especially applicable for screening tests of various compounds, e.g., antitumour drugs showing promising results in in vitro studies, before animal experiments. It was demonstrated that tumours grafted on the CAM surface had similar characteristics to the tumours grown in mammalian models, with the additional advantage that the setup of the CAM for cancer studies was faster [31]. Furthermore the CAM has been demonstrated as an excellent platform to test multiple drug delivery system formulations [32]. However, disadvantages such as unfeasible long-term evaluations, differences between avian metabolism and mammalian immune systems should also be considered. As a result, the higher efficiency of Au-GSH-Dox than free Dox in FISS treatment demonstrated in our study needs to be further validated in tumour-bearing mice. To perform further clinical trials on cats, the antitumour efficacy of intratumourally applied Au-GSH-Dox needs to be significantly higher than that of intravenously applied Au-GSH-Dox and intravenously and intratumourally applied free Dox. Moreover, further studies on rodent models to assess the pharmacokinetics of Dox after the intratumourally injected Au-GSH-Dox complex are needed. Systemic toxicity of intratumourally applied Au-GSH-Dox can be expected to be lower than that of intravenously applied Au-GSH-Dox, which could be one of the main advantages of intratumoural chemotherapy, however, this should be confirmed in toxicology tests using at least two different animal models. The presented model seems to be useful as a fast screening model for various novel compounds showing promising results in in vitro tests, that are dedicated to the further in vivo studies. Ki-67 is a proliferation marker which acts as a prognostic marker, e.g., for human mammary gland tumours and can be used to predict the effect of chemotherapy treatment. High Ki-67 expression may be an indicator of a good response to chemotherapy, but at the same time may worsen the prognosis. On the other hand, in the case of aggressive mammary gland tumours, lowering Ki-67 expression by 1% after chemotherapy treatment prolongs disease-free survival time [33]. There are only a few studies on the use of the Ki-67 proliferation marker for FISS. Eckstein et al. [34] showed that Ki-67 expression of FISS was between 10% and 40%. They also indicated that Ki-67 should not be used as a predictive factor for FISS, which is in agreement with the results of the presented study, in which we demonstrated no Ki-67 expression or lack of statistically important changes in Ki-67 expression after injections of tested compounds (Figure 4).

The limitations of the presented study should also be indicated. As doxorubicin in Au-GSH-Dox is non-covalently attached to gold nanoparticles, it is less stable than covalently attached doxorubicin. As a result, intratumoural injections (not intravenous injections) are recommended, as the drug may release gold nanoparticles into the bloodstream before targeting the tumour. Au-GSH-Dox described in this article may probably be used only for cutaneous cancer due to the ease of access for intratumoural injections.

## 3. Materials and Methods

### 3.1. Cell Culture

The FFS1 and FFS3 cell lines were obtained from Justus Liebig Universitat (Giessen, Germany) [35]. Cells were cultured in a 5% CO_2_ incubator with high glucose (4500 g/L) Dulbecco’s Modified Eagle Meddium (Gibco BRL, Life Technologies, Paisley, UK) enriched with heat-inactivated fetal bovine serum (FBS), penicillin-streptomycin (50 mL IU-1) and amphotericin B (2.5 mg·mL^−1^). The cells were trypsinized when their confluence reached 70%–80%.

### 3.2. Au-GSH-Dox Complex Preparation

Au-GSH-Dox complex was prepared according to the procedures described in our recent paper [9]. The bioconjugation protocol used non-covalent modes of binding based on a combination of electrostatic and hydrophobic interactions of the small peptide (glutathione) and doxorubicin salt. The non-covalent method was used for ligand conjugation to overcome problems associated with covalent conjugation methods. Briefly, the starting material was gold nanoparticles with glutathione molecules covalently attached to the surface via mercapto moieties. These nanoparticles were obtained using a slightly modified method published by Jin and co-workers [36] and purified from excess glutathione. The solution of glutathione-coated nanoparticles was then mixed with doxorubicin hydrochloride to obtain a non-covalent conjugate. In the last step purification of the resultant conjugate was performed by centrifugation in a centrifuge filter. The retentate was rinsed with phosphate buffer until the concentration of free doxorubicin hydrochloride was undetectable in the permeate. Nanoparticle samples were analysed using a wide range of available techniques e.g., TEM, SEM, XPS, SAXS to determine chemical composition and structural characteristics [9]. It is important to note, because of its versatility, that this method can be used for attachment of other drug molecules ligands and may serve as a universal strategy for ligand conjugation, such as targeting molecules or smart peptides.

### 3.3. CAM Assay

CAM assay was performed according to the previously described procedure [5,31]. In summary, eighty four Ross 308 fertilized chicken eggs (Pankowski Jan, Białobrzegi, Poland) were used. According to the Polish animal law (The Act on the Protection of Animals Used for Scientific and Educational Purposes was passed in January 2015 and replaced Directive 2010/63/EU in current Polish legislation), avian embryos are not considered as “live vertebrae animals”, so the Approval of Animal Ethics Commission was not required. On the 6th day of the chick embryo incubation 5 × 10^6^ FFS1 and FFS3 cells were injected into silicon rings, which had been put on the chorioallantoic membrane. On the 16th day of the chick embryo incubation tumours were observed in sixty four eggs (73%). Chick embryos with tumours both from FFS1 and FFS3 cell lines were randomly divided into four groups (seven eggs per group) and 20 μL of tested substances were injected intratumourally, into the concentrations obtained previously for Au-GSH-Dox in the MTT assay: 2.8 and 2.6 µg/mL (for the FFS1 and FFS3 cell line, respectively) [9], using 11G tuberculin syringes, as described:

1. Saline (negative control)
2. Au-GSH
3. Dox
4. Au-GSH-Dox

On the 16th and 19th day of the chick embryo incubation the tumours were photographed with a Digital MacroView™ Otoscope (WelchAllyn, Skaneateles Falls, NY, USA) and their size was measured with computer software. On the 19th day of incubation the chick embryos were sacrificed by spinal cord dislocation, tumours were removed from eggs and measured once more with a calliper. Two tumours were eliminated from the study as the sizes obtained on the last day with the computer software and the calliper did not correlate with each other (only small parts of the tumours were visible with the digital otoscope). Tumour volume (V) was measured according to the formula: V = 4/3πr^3^ (r: radius)l, as their shape was oval. Tumour volume ratios (before and after adding tested compounds) were calculated for each tumour.

### 3.4. Immunohistochemistry

Histopathological staining for Ki-67 (clone MIB 1, dilution 1:75 in 1% bovine serum) (Dako, Glostrup, Denmark) was performed for each tumour according to the manufacturer’s protocol to assess the influence of tested compounds on the Ki-67 proliferation marker. Paraffin-embedded tissues of canine mammary gland tumors were used as positive controls [37]. Negative control was done by omitting primary antibodies. The pictures were taken using an Olympus BX60 microscope (Olympus, Hamburg, Germany). Ki-67 labeling index (LI) was defined as a percentage of positively stained tumour cells (brown reaction in the cell nuclei) among the total number of malignant cells assessed [38,39] and presented as mean with standard deviation (SD). Necrotic areas and all inflammatory cells were omitted.

### 3.5. Statistical Analysis

One-way ANOVA and Tukey’s Honestly Significant Difference (HSD) post-hoc test were performed (GraphPad Prism version 5.00 for Windows, GraphPad Software, San Diego, CA, USA). *p* < 0.05 was assigned as significant, while *p* < 0.01 as highly significant.

## 4. Conclusions

The volume ratio of tumours grown on the CAM from the FFS1 and FFS3 cell lines was significantly (*p* < 0.01) decreased after a single intratumoural injection of Au-GSH-Dox, which confirms the positive results of in vitro studies and indicates that Au-GSH-Dox may be a potent new therapeutic agent for feline injection-site sarcomas. Nevertheless, the results demonstrated in our study need to be further validated in tumour-bearing mice.
